# Concurrent dose-finding of a novel cancer drug with and without a second agent

**DOI:** 10.1017/cts.2023.542

**Published:** 2023-05-10

**Authors:** Nolan A. Wages, Ramy R. Saleh, Thomas M. Braun

**Affiliations:** 1 Department of Biostatistics, School of Medicine, Virginia Commonwealth University, Richmond, VA, USA; 2 Massey Cancer Center, Virginia Commonwealth University, Richmond, VA, USA; 3 Division of Medical Oncology & Hematology, Department of Medicine, Princess Margaret Cancer Centre, and the University of Toronto, Toronto, ON, Canada; 4 Department of Biostatistics, School of Public Health, University of Michigan, Ann Arbor, MI, USA

**Keywords:** Cancer, clinical trial design, dose-finding, drug combination, shift model

## Abstract

**Introduction:**

More complex research questions are being posed in early-phase oncology clinical trials, necessitating design strategies tailored to contemporary study objectives. This paper describes the proposed design of a Phase I trial concurrently evaluating the safety of a hematopoietic progenitor kinase-1 inhibitor (Agent A) as a single agent and in combination with an anti-PD-1 agent in patients with advanced malignancies. The study’s primary objective was to concurrently determine the maximum tolerated dose (MTD) of Agent A with and without anti-PD-1 therapy among seven possible study dose levels.

**Methods:**

Our solution to this challenge was to apply a continual reassessment method shift model to meet the research objectives of the study.

**Results:**

The application of this method is described herein, and a simulation study of the design’s operating characteristics is conducted. This work was developed through collaboration and mentoring between the authors at the American Association for Cancer Research (AACR) and the American Society of Clinical Oncology (ASCO) annual AACR/ASCO Methods in Clinical Cancer Research Workshop.

**Conclusions:**

The aim of this manuscript is to highlight examples of novel design applications as a means of augmenting the implementation of innovative designs in the future and to demonstrate the flexibility of adaptive designs in satisfying modern design conditions. Although the design is presented using an investigation of Agent A with and without anti-PD-1 therapy as an illustrative example, the approach described is not specific to these agents and could be applied to other concurrent monotherapy and combination therapy studies with well-defined binary safety endpoints.

## Introduction

The paper describes the proposed design of a Phase I trial concurrently evaluating the safety of a hematopoietic progenitor kinase-1 inhibitor (Agent A) as a single agent and in combination with an anti-PD-1 agent in patients with advanced malignancies. This work was developed through collaboration and mentoring between the authors at the American Association for Cancer Research (AACR) and the American Society of Clinical Oncology (ASCO) annual AACR/ASCO Methods in Clinical Cancer Research Workshop. The study’s primary objective was to concurrently determine the maximum tolerated dose (MTD) of Agent A with and without anti-PD-1 therapy, among the possible study dose levels provided in Table 1. The starting dose level was 60 mg once daily. The MTD in each row of Table 1 was defined as the study dose level with a dose-limiting toxicity (DLT) rate closest to the prespecified target DLT rate of 30%. In this trial, several assumptions drive the design considerations. First, the DLT probability increases as Agent A’s dose increases (i.e., across rows of Table 1). Second, the addition of anti-PD-1 is accompanied by additional DLT-qualifying adverse events to those related to Agent A which increases the likelihood of DLT of the combination therapy relative to the monotherapy when holding the dose of Agent A constant (i.e., up columns of Table 1). Therefore, before the study, clinical information would indicate that the estimated MTD of Agent A without anti-PD1 therapy should not be below the estimated DLT with anti-PD-1 treatment.

**Table 1.** Study dose levels of a Phase I trial a hematopoietic progenitor kinase-1 inhibitor (Agent A) as a single agent and in combination with an anti-PD-1 agent

	Dose (mg)						
Agent A	60	120	240	480	800	1200	1600
PD-1 (yes)							
PD-1 (no)							

A straightforward way to design this study would be to conduct parallel dose-finding trials to identify the MTD of Agent A both with and without anti-PD-1 therapy. In this case, a method intended for single-agent evaluation, such as the 3 + 3 algorithm, could be applied independently to each row of Table 1. Conducting independent trials for each row is a simple approach to the problem described, but it is a suboptimal way to proceed because doing so ignores the available clinical information between the two treatment strategies. This approach often leads to a reversal, a situation in which MTD recommendations violate the known order of toxicity risk by row [1]. Reversals are problematic because the final MTD selection for Agent A with anti-PD-1 therapy is a higher dose level than the MTD for Agent A without anti-PD-1 treatment, which is assumed to be less toxic. In this study, either the MTD in each row should be at the same dose of Agent A or the MTD with anti-PD-1 should be below the MTD without Agent A. A more efficient approach would involve adaptively borrowing information across rows of Table 1 to allow safety data from Agent A with ant-PD-1 therapy to inform dose assignments for Agent A without anti-PD-1 treatment and vice versa.

Several existing dose-finding methods for drug combinations could be applied to this problem. The sequential continual reassessment method (CRM [2]) proposed by Yin and Yuan [3] converts the two-dimensional dose-finding trial to a series of one-dimensional dose-finding subtrials by fixing the dose level of one drug, but they suggest conducting the subtrials sequentially. An adaptive approach to this problem was developed by Braun and Jia [4]. However, neither method has available software for simulation or implementation. Ivanova and Wang [5] applied bivariate isotonic regression to estimate multiple MTD in drug combination studies. An editorial in the *Journal of Clinical Oncology* by Mandrekar [6] described the use of the method of Ivanova and Wang [5] in a real phase I study aiming to identify multiple MTD [7]. This method also does not have accompanying software for simulation or implementation. The product of independent beta priors design, proposed by Mander *et al*. [8], enables the estimation of a maximum tolerated contour, a set of dose combinations with acceptable toxicity profiles. The product of independent priors design has an R package (pipe.design) available for download on the Comprehensive R Archive Network and accompanying R shiny web application. However, this method is not explicitly geared toward finding an MTD combination in each row of a two-dimensional grid. It may recommend any number (> 2) MTD combinations for further testing in middle development. The two-dimensional design of Wages [9] has demonstrated good statistical properties compared to the Wang and Ivanova [5] method. The method has available R code for simulation upon request from the first author. We will use this method to illustrate the advantages of using a design that efficiently borrows safety data across the dose levels of Agent A with and without the addition of anti-PD1 therapy. The statistical properties of such an approach will be contrasted with a framework that conducts independent parallel trials using a design for single-agent evaluation, such as 3 + 3 decision rules or a Bayesian optimal interval (BOIN) method [10], in each row.

## Methods

A participant is classified as experiencing DLT based on protocol-specific adverse event definitions occurring during the first treatment cycle. As data accumulate, each participant is classified as to whether they experienced a DLT (yes/no). Adverse events were to be graded using the National Cancer Institute (NCI) Common Terminology Criteria (CTCAE) Version 5.0. The MTD of Agent A in each row of Table 1 is defined as the dose combination with a DLT rate closest to the target rate of 30%.

It is assumed that the MTD for Agent A in each row is either (i) at the same dose of the anti-PD-1 therapy, or (ii) the MTD for Agent A is one dose level lower with the addition of anti-PD-1 therapy than without the anti-PD-1 treatment. For instance, if the MTD dose of Agent A without anti-PD-1 therapy is estimated to be dose level 4 (480 mg). The estimated MTD of Agent A with anti-PD-1 treatment will also be dose level 4 (480 mg) or will be *shifted* one level lower to dose level 3 (240 mg). The truth could be either of these possibilities. We want to account for this uncertainty in the design by using the data to estimate the relative location of the MTD between rows. A similar strategy has been implemented in methods that account for patient heterogeneity [11,12]. The relative location of the MTD between rows is illustrated in Table 2.

**Table 2.** Illustration of the relative location between maximum tolerated doses in each row of a drug combination grid. Colored cells indicate the hypothesized MTD in each row

Doses of Agent A	Maximum tolerated doses are the same in each row						
	60 mg	120 mg	240 mg	480 mg	800 mg	1200 mg	1600 mg
PD-1 (+)				MTD_+_			
PD-1 (−)				MTD_-_			

Doses of Agent A	Maximum tolerated doses differ by one dose level						
	60 mg	120 mg	240 mg	480 mg	800 mg	1200 mg	1600 mg
PD-1 (+)			MTD_+_				
PD-1 (−)				MTD_-_			

MTD, maximum tolerated dose.

### Estimation and Allocation

The design is based on utilizing a class of working models corresponding to relative locations (shifts) of the MTD in each row of the drug combination grid (Table 2). The uncertainty associated with the relationship between the MTD in each row is expressed through the specification of multiple one-parameter models in Table 3 that reflect different locations of the MTD of Agent A with and without anti-PD-1 therapy. Model selection techniques are used to choose the model most consistent with the observed data. A common model choice [13] in the CRM is to raise a set of initial DLT probability estimates, also referred to as the “skeleton” of the model, to a power *a* that is a parameter to be estimated by the data. A different skeleton is specified for each possible MTD shift between the dose levels (i.e., shift = 0 or shift = −1). The skeleton values displayed in Table 3 for each model were generated using the algorithm of Lee and Cheung [14]. Using all accumulated toxicity data, the design fits the CRM for each DLT probability working model, and the parameter *a* is estimated for each model by maximum likelihood estimation. The working model with the largest likelihood is chosen, and DLT probability estimates are updated for each dose level using the selected model. If there is a tie between the highest likelihood values of two or more models, then the selected model is randomly chosen from among those with tied likelihood values. The updated DLT probability estimates are used to identify the dose of Agent A in each row with a DLT rate closest to the target rate of 30%. The design would then randomize the next patient to one of the two recommended dose levels with equal probability.

**Table 3.** Working models for the dose-limiting toxicity probabilities at each dose level. Colored cells indicate the hypothesized MTD in each row

	Working model 1 (maximum tolerated doses are the same in each row)						
*m* = 1 (shift = 0)	0.06^exp(a)^	0.12 ^exp(a)^	0.20 ^exp(a)^	0.30 ^exp(a)^	0.40 ^exp(a)^	0.50 ^exp(a)^	0.59 ^exp(a)^
	0.06 ^exp(a)^	0.12 ^exp(a)^	0.20 ^exp(a)^	0.30 ^exp(a)^	0.40 ^exp(a)^	0.50 ^exp(a)^	0.59 ^exp(a)^

	Working model 2 (maximum tolerated doses differ by one level)						
*m* = 2 (shift = −1)	0.12 ^exp(a)^	0.20 ^exp(a)^	0.30 ^exp(a)^	0.40 ^exp(a)^	0.50 ^exp(a)^	0.59 ^exp(a)^	0.67 ^exp(a)^
	0.06 ^exp(a)^	0.12 ^exp(a)^	0.20 ^exp(a)^	0.30 ^exp(a)^	0.40 ^exp(a)^	0.50 ^exp(a)^	0.59 ^exp(a)^

MTD, maximum tolerated dose.

### Getting the Trial Underway

Likelihood-based methods, like the two-stage CRM, fail to have a solution until at least one DLT and one non-DLT have been observed in the study [15]. Thus, an initial dose escalation scheme must be defined before the study begins that will be used until one DLT and one non-DLT occur in the study. This initial design essentially amounts to prespecifying a path within the drug combination grid to adhere to get the trial underway. This process could, of course, be carried out in several ways and could include some randomization if more than one dose level could be considered for escalation, as is often the case in drug combination studies. In this design, the trial would start at the lowest dose level and escalate along the bottom row in cohorts of size one until the first DLT occurs. The design is flexible enough to accommodate other starting dose levels if necessitated in a specific trial situation. As long as no DLT is observed in participants not assigned to anti-PD-1, escalation would continue at the lowest dose level of Agent A with anti-PD-1 therapy. After the first DLT, the modeling stage begins, which proceeds according to the estimation and allocation procedure described above.

## Results

### Illustration of the Method

In this section, we illustrate the behavior of the method described in this article under a set of true DLT probabilities for the 2 × 7 example described in Section 2. The target DLT rate is 30%, and the total sample size is *N* = 39, which was chosen using the sample size calculator for CRM trials [16] using the function **getn** in R package **dfcrm [** 17] with the following specifications; (1) the desired average probability of correction selection (PCS) of 50%, (2) target DLT rate of 30%, (3) the number of test doses equal to 14 (i.e., 7 dose levels in 2 rows), and (4) effect size equal to 1.78. The set of true DLT probabilities for row 1 are {0.01, 0.12, 0.18, 0.21, 0.22, **0.31**, 0.60}, indicating that dose level 6 (i.e., Agent A at 1200mg *without* a-PD-1 therapy) is the true MTD in row 1. For row 2, the true probabilities are {0.09, 0.14, 0.22, 0.25, **0.32**, 0.40, 0.64}, indicating that dose level 5 (i.e., Agent A at 800 mg *with* a-PD-1 therapy) is the true MTD in row 2. The relative location of these true MTD combinations indicates a shift of one dose level between the two rows. The method embodies the characteristics of the CRM, so we appeal to its features in specifying design parameters. The skeleton values were chosen by the algorithm of Lee and Cheung [14] and are provided in Table 3. We assumed that each working model was equally likely to represent the true shift between the MTD combinations at the beginning of the trial.

The data from the entire simulated trial are provided in Table 4. The first four eligible participants are administered escalating dose levels along row 1, and 0 DLT are observed on doses of 60, 120, 240, and 480 mg of Agent A without anti-PD-1 therapy. The first DLT occurs in participant 5 on a dose of 800 mg of Agent A without anti-PD-1, at which point the modeling stage begins. Even with these limited data, the true shift between MTD combinations is estimated to be one dose level apart. Combinations (480 mg with a-PD-1 therapy) and (800 mg without a-PD-1 therapy) are indicated to have an estimated DLT rate closest to the target rate. Patient 6 is randomized with probability 1/2 to one of these combinations, which yields a recommendation of (480 mg with anti-PD-1 therapy), on which they do not experience DLT. It is important to note that the dose-limiting probabilities are updated in row 2, even though we have yet to observe a patient in this row, illustrating the formal borrowing of information across rows afforded by the model. Overall, in this simulated trial (Table 4), *N* = 39 patients were treated, yielding final MTD combination recommendations of (800 mg with anti-PD-1 therapy) and (1200 mg without anti-PD-1 treatment).

**Table 4.** Illustration of proposed method

Pt	Dose of A	a-PD-1 (±)	DLT?	Est shift	Pt	Dose of A	a-PD1 (±)	DLT?	Est shift
1	60	–	No	n/a	21	1200	–	No	−1
2	120	–	No	n/a	22	1200	–	No	−1
3	240	–	No	n/a	23	800	+	No	−1
4	480	–	No	n/a	24	800	+	No	−1
5	800	–	Yes	−1	25	1200	–	No	−1
6	480	+	No	0	26	1200	–	No	−1
7	800	–	No	0	27	1200	+	Yes	−1
8	1200	–	No	0	28	800	–	Yes	−1
9	1200	+	No	0	29	1200	–	No	−1
10	1600	+	Yes	0	30	1200	–	No	−1
11	1200	+	Yes	−1	31	1200	–	No	−1
12	800	+	No	−1	32	1200	–	Yes	−1
13	800	+	No	0	33	1200	–	No	−1
14	1200	–	No	−1	34	1200	–	Yes	−1
15	800	+	No	0	35	1200	–	No	−1
16	1200	+	Yes	−1	36	800	+	No	−1
17	800	+	Yes	−1	37	800	+	No	−1
18	1200	–	No	−1	38	800	+	No	−1
19	800	+	No	−1	39	1200	–	No	−1
20	800	+	Yes	−1					
					MTD_+_ = 800		MTD_−_ = 1200		

DLT, dose-limiting toxicity; MTD, maximum tolerated dose.

### Operating Characteristics Over a Small Set of Curves

We compared the operating characteristics of three competing approaches for identifying MTD of Agent A both with and without anti-PD-1 therapy. The first approach is to run independent 3 + 3 algorithms in each row. The second approach is to run independent BOIN designs in each row. The third approach was the CRM design for identifying multiple MTD combinations using a shift model described above.

The operating characteristics of the three methods were compared by simulating 1000 trials under six dose-toxicity cases of a 2 × 7 dose combination grid with varying positions of actual MTDs, as shown in Table 5. We randomly generated the dose–toxicity curves using the method of Conaway and Petroni [18], with the constraint that the MTD in each row are the same or one level apart. The target DLT rate that defines the MTD is set to 30% in all scenarios. The sample size is *N* = 39 for the CRM shift model design, and *n* = 20 in each row (total *N* = 40) for the independent BOIN designs. The sample size for the two separate 3 + 3 trials is estimated from the simulation studies. Throughout the simulation studies, participants are assigned to doses in cohorts of size one in the independent BOIN and shift model approaches. In the specification of our working models, we restrict the possible shift between MTD to be either 0 or −1, indicating that we do not allow for more than a one-dose-level shift between two adjacent rows of the matrix; these shifts represent the clinical setting in which we are operating. However, shifts of two or more are possible, and the CRM shift model can handle such possibilities by including more working models, as demonstrated in Wages [9]. In general, our goal is to evaluate (i) how well each method locates MTD at and around the target DLT rate in each row (percentage of correct recommendation; PCR) and (ii) how well each method allocates patients to combinations at and around the target DLT rate in each row (proportion of correct allocation; PCA), and (iii) how many times did the independent designs “reverse” the order of the MTD (i.e., chooses a higher MTD with anti-PD-1 than without anti-PD-1)? This percentage will be 0% for the CRM design since the shift model structure prevents it from happening, and thus it is not possible.

**Table 5.** Percent of maximum tolerated dose selection, percent of trials that stopped early for safety, and the percentage of trials that resulted in a reversal for each of the three approaches. Colored cells indicate the hypothesized MTD in each row

		Hypothesized probability of dose-limiting toxicity at each treatment combination.							% trials w/a reversal	% stop early
Case 1	PD-1 (+)	0.09	0.14	0.22	0.25	0.32	0.40	0.64		
	PD-1 (−)	0.01	0.12	0.18	0.21	0.22	0.31	0.60		
3 + 3	PD-1 (+)	15.3	27.3	19.9	15.0	9.2	4.7	0.3	29.2%	6.5%
	PD-1 (−)	11.6	21.9	20.2	15.3	15.5	14.1	1.2		0.0%
BOIN	PD-1 (+)	5.3	13.2	17.4	21.7	20.3	17.7	2.0	29.3%	2.4%
	PD-1 (−)	4.2	10.2	12.1	13.2	21.6	33.1	5.5		0.1%
Shift	PD-1 (+)	0.0	2.4	10.4	26.7	40.0	20.3	0.2	0%	0%
	PD-1 (−)	0.0	0.2	6.6	16.3	31.9	40.5	4.5		
Case 2	PD-1 (+)	0.20	0.24	0.31	0.43	0.56	0.65	0.72		
	PD-1 (−)	0.01	0.05	0.09	0.31	0.45	0.55	0.68		
3 + 3	PD-1 (+)	26.1	23.8	15.2	6.1	0.9	0.1	0.0	6.5%	29.9%
	PD-1 (−)	2.3	8.4	45.3	32.7	9.9	1.2	0.2		0.2%
BOIN	PD-1 (+)	18.0	23.5	27.6	15.4	3.6	1.1	0.2	10.7	10.6%
	PD-1 (−)	3.2	3.8	24.3	44.8	16.9	5.9	1.1		0.0%
Shift	PD-1 (+)	0.4	14.2	52.2	29.7	3.5	0.0	0.0	0%	0%
	PD-1 (−)	0.0	0.7	18.6	63.3	17.0	0.4	0.0		
Case 3	PD-1 (+)	0.34	0.41	0.45	0.57	0.65	0.69	0.79		
	PD-1 (−)	0.26	0.40	0.43	0.52	0.55	0.60	0.71		
3 + 3	PD-1 (+)	27.2	10.6	2.3	0.2	0.0	0.0	0.0	22.1%	59.3%
	PD-1 (−)	40.5	12.8	4.3	0.7	0.1	0.0	0.0		44.1%
BOIN	PD-1 (+)	32.6	15.7	9.2	2.0	0.6	0.2	0.0	28.3	39.7%
	PD-1 (−)	38.9	19.3	11.7	5.4	1.2	0.5	0.0		23.1%
Shift	PD-1 (+)	67.6	17.6	4.6	0.3	0.0	0.0	0.0	0%	9.9%
	PD-1 (−)	48.9	29.1	10.7	1.3	0.1	0.0	0.0		
Case 4	PD-1 (+)	0.03	0.08	0.15	0.33	0.41	0.85	0.88		
	PD-1 (−)	0.01	0.04	0.07	0.32	0.40	0.82	0.85		
3 + 3	PD-1 (+)	6.5	19.4	41.6	22.0	9.1	0.0	0.0	22.0%	0.7%
	PD-1 (−)	1.3	4.6	50.0	31.2	12.9	0.0	0.0		0.2%
BOIN	PD-1 (+)	3.2	6.5	31.8	36.0	21.8	0.6	0.0	31.3%	0.1%
	PD-1 (−)	3.7	3.5	25.2	41.6	25.3	0.7	0.0		0.0
Shift	PD-1 (+)	0.0	1.1	26.1	57.2	15.4	0.2	0.0	0.0%	0.0%
	PD-1 (−)	0.0	0.0	9.3	57.0	32.9	0.8	0.0		
Case 5	PD-1 (+)	0.11	0.16	0.32	0.37	0.47	0.51	0.74		
	PD-1 (−)	0.06	0.14	0.18	0.32	0.44	0.44	0.70		
3 + 3	PD-1 (+)	19.1	40.0	19.3	8.5	2.6	0.2	0.0	23.3%	10.5%
	PD-1 (−)	16.1	18.9	33.6	22.0	4.8	1.4	0.0		2.7%
BOIN	PD-1 (+)	8.5	29.7	26.9	19.6	8.9	3.8	0.2	24.7%	2.4%
	PD-1 (−)	5.0	11.8	25.1	32.4	13.5	10.0	1.1		1.1%
Shift	PD-1 (+)	0.9	13.6	47.0	29.4	7.7	1.4	0.0	0%	0%
	PD-1 (−)	0.1	2.6	28.6	48.1	16.9	3.3	0.4		
Case 6	PD-1 (+)	0.07	0.08	0.16	0.21	0.31	0.50	0.60		
	PD-1 (−)	0.06	0.08	0.10	0.20	0.27	0.43	0.54		
3 + 3	PD-1 (+)	5.5	17.8	21.5	26.8	19.8	3.4	0.3	35.2%	4.2%
	PD-1 (−)	4.8	10.7	25.2	27.1	22.2	6.2	0.6		4.0%
BOIN	PD-1 (+)	3.8	6.7	14.3	26.8	32.0	12.9	2.4	32.6%	1.1%
	PD-1 (−)	2.4	4.9	10.9	23.7	29.9	22.3	5.0		0.9%
Shift	PD-1 (+)	0.1	0.5	7.0	32.3	47.3	12.2	0.5	0.0%	0.1%
	PD-1 (−)	0.0	0.1	2.1	19.0	46.8	29.2	2.7		

BOIN, Bayesian optimal interval; MTD, maximum tolerated dose.

Tables 5 and 6 show the operating characteristics of the three methods under the six dose-toxicity cases. Table 7 reports the proportion of trials correctly identifying neither, one, or both MTDs. In case I, the shift model method yields a higher PCR than the parallel BOIN and 3 + 3 approaches in both of the rows (40.5% vs. 33.1% vs. 14.1% in row 1; 40.0% vs. 20.3% vs. 9.2% in row 2) and an average PCR of 40.25% across both rows, compared with 11.65% for the independent 3 + 3 approach and 26.7% for the parallel BOIN approach. For patient allocation, the proposed method allocates the highest proportion of patients to the true MTD in both of the rows in case I (0.14 vs. 0.10 vs. 0.04 in row 1; 0.13 vs. 0.08 vs. 0.04 in row 2) and yields the highest overall PCA (0.27) than parallel 3 + 3 and BOIN algorithms (0.08 and 0.10, respectively). The average sample size is 31.8 for the parallel 3 + 3 design, 39.6 for the parallel BOIN designs, and 39.0 for the shift model approach in case I – the parallel 3 + 3 approach results in a reversal in 29.2% of simulated trials and the parallel BOIN approach does so in 29.3% of simulated trials.

**Table 6.** Proportion of patient allocation and sample size for each of the three approaches. Colored cells indicate the hypothesized MTD in each row

		Hypothesized probability of dose-limiting toxicity at each treatment combination.							Sample size by row	Total sample size
Case 1	PD-1 (+)	0.09	0.14	0.22	0.25	0.32	0.40	0.64		
	PD-1 (−)	0.01	0.12	0.18	0.21	0.22	0.31	0.60		
3 + 3	PD-1 (+)	0.12	0.11	0.10	0.07	0.04	0.02	0.01	14.7	31.8
	PD-1 (−)	0.10	0.12	0.11	0.09	0.06	0.04	0.02	17.1	
BOIN	PD-1 (+)	0.06	0.09	0.09	0.09	0.08	0.06	0.03	19.6	39.6
	PD-1 (−)	0.05	0.08	0.08	0.08	0.08	0.10	0.05	20.0	
Shift	PD-1 (+)	0.02	0.03	0.06	0.11	0.13	0.08	0.02	17.5	39.0
	PD-1 (−)	0.03	0.04	0.07	0.10	0.13	0.14	0.05	21.5	
Case 2	PD-1 (+)	0.20	0.24	0.31	0.43	0.56	0.65	0.72		
	PD-1 (−)	0.01	0.05	0.09	0.31	0.45	0.55	0.68		
3 + 3	PD-1 (+)	0.16	0.11	0.07	0.03	0.01	0.00	0.00	10.5	26.9
	PD-1 (−)	0.12	0.13	0.13	0.14	0.07	0.02	0.00	16.4	
BOIN	PD-1 (+)	0.12	0.12	0.12	0.08	0.03	0.01	0.00	18.1	38.1
	PD-1 (−)	0.03	0.05	0.14	0.17	0.09	0.04	0.01	20.0	
Shift	PD-1 (+)	0.02	0.07	0.16	0.13	0.05	0.01	0.00	17.2	39.0
	PD-1 (−)	0.03	0.04	0.12	0.22	0.11	0.03	0.01	21.8	
Case 3	PD-1 (+)	0.34	0.41	0.45	0.57	0.65	0.69	0.79		
	PD-1 (−)	0.26	0.40	0.43	0.52	0.55	0.60	0.71		
3 + 3	PD-1 (+)	0.30	0.12	0.03	0.01	0.00	0.00	0.00	6.7	14.5
	PD-1 (−)	0.29	0.17	0.06	0.01	0.00	0.00	0.00	7.8	
BOIN	PD-1 (+)	0.23	0.12	0.07	0.03	0.01	0.00	0.00	14.7	31.6
	PD-1 (−)	0.25	0.14	0.08	0.04	0.02	0.01	0.00	16.9	
Shift	PD-1 (+)	0.31	0.09	0.04	0.02	0.01	0.0	0.0	17.1	36.7
	PD-1 (−)	0.28	0.14	0.08	0.03	0.01	0.0	0.0	19.6	
Case 4	PD-1 (+)	0.03	0.08	0.15	0.33	0.41	0.85	0.88		
	PD-1 (−)	0.01	0.04	0.07	0.32	0.40	0.82	0.85		
3 + 3	PD-1 (+)	0.10	0.11	0.12	0.10	0.04	0.01	0.00	15.2	31.4
	PD-1 (−)	0.10	0.11	0.11	0.13	0.06	0.01	0.00	16.2	
BOIN	PD-1 (+)	0.04	0.07	0.14	0.14	0.10	0.03	0.00	20.0	40.0
	PD-1 (−)	0.03	0.04	0.14	0.15	0.11	0.03	0.00	20.0	
Shift	PD-1 (+)	0.00	0.02	0.12	0.20	0.09	0.01	0.00	17.1	39.0
	PD-1 (−)	0.03	0.03	0.08	0.22	0.17	0.03	0.00	21.9	
Case 5	PD-1 (+)	0.11	0.16	0.32	0.37	0.47	0.51	0.74		
	PD-1 (−)	0.06	0.14	0.18	0.32	0.44	0.44	0.70		
3 + 3	PD-1 (+)	0.14	0.13	0.11	0.05	0.02	0.00	0.00	12.4	27.1
	PD-1 (−)	0.13	0.14	0.12	0.10	0.04	0.01	0.00	14.7	
BOIN	PD-1 (+)	0.07	0.14	0.12	0.08	0.05	0.02	0.01	19.6	39.4
	PD-1 (−)	0.06	0.08	0.12	0.12	0.07	0.04	0.02	19.8	
Shift	PD-1 (+)	0.03	0.07	0.15	0.12	0.06	0.02	0.00	17.5	39.0
	PD-1 (−)	0.04	0.06	0.13	0.17	0.10	0.04	0.01	21.5	
Case 6	PD-1 (+)	0.07	0.08	0.16	0.21	0.31	0.50	0.60		
	PD-1 (−)	0.06	0.08	0.10	0.20	0.27	0.43	0.54		
3 + 3	PD-1 (+)	0.10	0.10	0.10	0.09	0.06	0.03	0.00	16.8	34.4
	PD-1 (−)	0.10	0.10	0.10	0.10	0.07	0.04	0.01	17.6	
BOIN	PD-1 (+)	0.04	0.06	0.09	0.11	0.11	0.06	0.02	19.8	39.6
	PD-1 (−)	0.04	0.05	0.08	0.10	0.11	0.08	0.04	19.8	
Shift	PD-1 (+)	0.01	0.02	0.05	0.13	0.15	0.06	0.02	17.2	39.0
	PD-1 (−)	0.03	0.03	0.05	0.11	0.18	0.12	0.04	21.8	

BOIN, Bayesian optimal interval; MTD, maximum tolerated dose.

**Table 7.** Percentage of trials that correctly recommend zero, one, and two maximum tolerated dose combinations for each method

	0			1			2		
Method	3 + 3	BOIN	Shift	3 + 3	BOIN	Shift	3 + 3	BOIN	Shift
Case 1	77.6%	54.3%	44.0%	21.5%	38.0%	31.5%	0.9%	7.7%	24.5%
Case 2	57.3%	39.8%	32.0%	37.5%	48.6%	20.5%	5.2%	11.6%	47.5%
Case 3	43.1%	40.3%	32.4%	46.1%	47.9%	18.7%	10.8%	11.8%	48.9%
Case 4	54.5%	36.3%	24.9%	37.8%	49.8%	36.0%	7.7%	13.9%	39.1%
Case 5	62.4%	50.0%	35.1%	33.9%	40.7%	34.7%	3.7%	9.3%	30.2%
Case 6	62.7%	48.0%	34.0%	32.6%	42.1%	37.9%	4.7%	9.9%	28.1%
Mean	59.6%	44.8%	33.7%	34.9%	44.5%	29.9%	5.5%	10.7%	36.4%

BOIN, Bayesian optimal interval.

In case 2, the shift model method yields a higher PCR than the parallel BOIN and 3 + 3 approaches in both of the rows (63.3% vs. 44.8% vs. 32.7% in row 1; 52.2% vs. 27.6% vs. 15.2% in row 2). The average PCR is 57.75% across all rows for the shift model approach, compared with 23.95% for the 3 + 3 approach and 36.2% for the BOIN approach. For patient allocation, the proposed method allocates the highest proportion of patients to the true MTD combinations in both of the rows in case 2 (0.22 vs. 0.17 vs. 0.14 in row 1; 0.16 vs. 0.12 vs. 0.07 in row 2) and yields a higher overall PCA (0.38) than parallel BOIN (0.29) and 3 + 3 algorithms (0.21). The average sample size is 26.9 for the parallel 3 + 3 design, 38.1 for the parallel BOIN design, and 39.0 for the shift model approach in case 2 – the parallel 3 + 3 approach results in a reversal in 6.5% of simulated trials and the BOIN approach does so in 10.7% of trials.

In case 3, the shift model method again produces a higher PCR than the parallel BOIN and 3 + 3 approaches in both of the rows (48.9% vs. 38.9% vs. 40.5% in row 1; 67.6% vs. 32.6% vs. 27.2% in row 2). The average PCR is 58.25% for the proposed approach across all rows, compared with 33.85% for the 3 + 3 approach and 38.9% for the BOIN approach. For patient allocation, the 3 + 3 and shift model methods allocate a similar proportion of patients to the true MTD combinations in both of the rows in case 3 (0.28 vs. 0.29 in row 1; 0.31 vs. 0.30 in row 2) and produce a similar overall PCA (0.59). The parallel BOIN approach allocates a slightly lower proportion of patients to true MTD combinations in each row. The average sample size is 14.5 for the parallel 3 + 3 design, 31.6 for the BOIN approach, and 36.7 for the shift model approach in case 1. This discrepancy is due to the aggressive nature in which the 3 + 3 stops early for safety concerns. Despite the safe doses in each row, the 3 + 3 stops early in 44.1% and 59.9% of simulated trials in rows 1 and 2, respectively. The parallel 3 + 3 approach results in a reversal in 22.1% of simulated trials and the parallel BOIN approach does so 28.3% of the time. Similar findings are reported for cases 4-6.

### Operating Characteristics Over Many Curves

**Figure 1.**
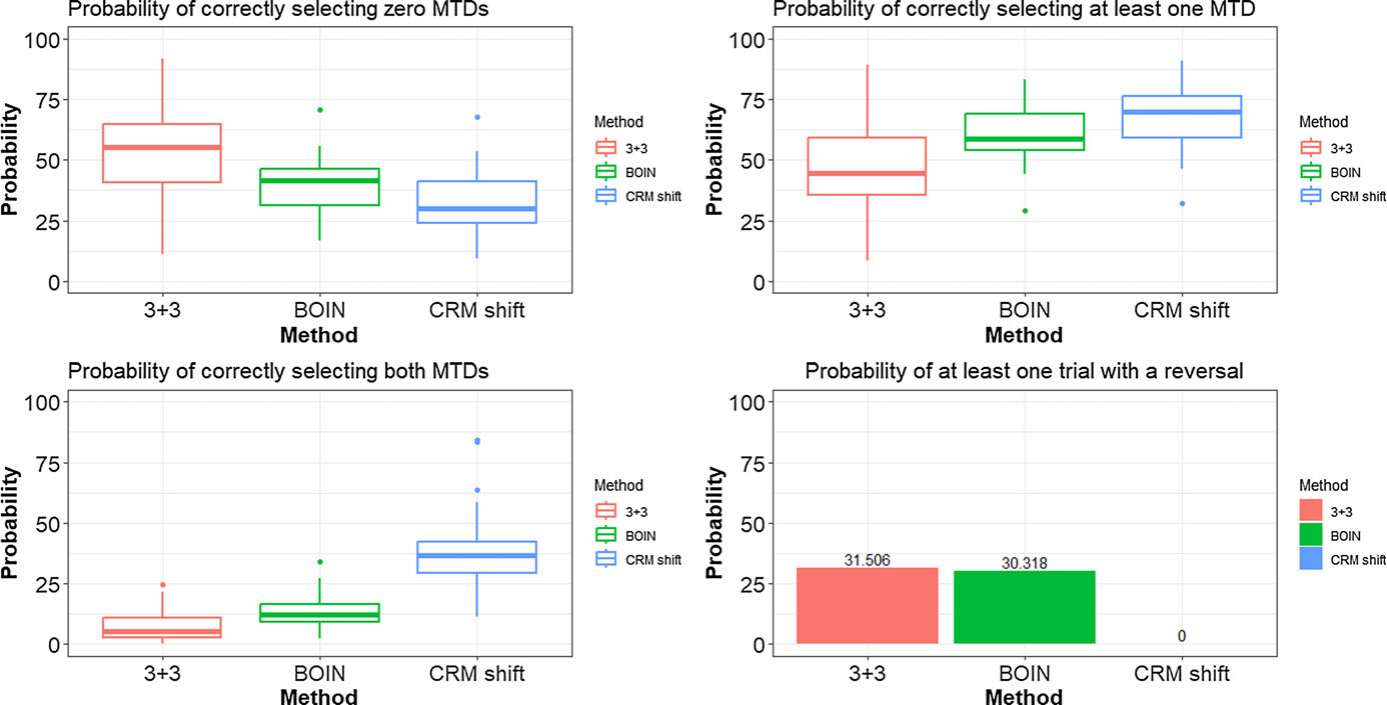
Operating characteristics of the three competing approaches over 50 randomly generated dose–toxicity curves. BOIN, Bayesian optimal interval; CRM, continual reassessment method; MTD, maximum tolerated dose.

We also simulated the operating characteristics of the three competing approaches over numerous randomly generated dose–toxicity curves. We randomly generated 50 dose–toxicity curves using the method of Conaway and Petroni [18], with the constraint that the MTDs in each row are the same or one level apart. The curves have a variety of actual MTD locations and shapes (i.e., steep, flat). We then simulated 1000 trials under each curve and evaluated the methods on four performance metrics reported in the previous section. First, how many times did each design correctly recommend neither of the true MTDs in each row (i.e., zero MTDs correct)? Second, how many times did each design correctly recommend at least one of the true MTDs in either row (i.e., at least one MTD correct)? Third, how many times did each design correctly recommend both true MTDs in each row (i.e., both MTD right)? Fourth is the percentage of reversals generated by the independent 3 + 3 design. This percentage will be 0% for the CRM design because the shift model structure prevents it from happening. We summarize the results of the 50 curves with boxplots for each of the first three metrics and a bar plot for the fourth metric (Fig. 1).

The average sample size, estimated from simulations, was approximately 26.9 for the independent 3 + 3 designs. The maximum sample size for the CRM shift model design and the parallel BOIN design was 39 and 40 participants, respectively. The probability of correctly selecting neither of the two true MTDs was 60% for the parallel 3 + 3 design, 40% for the parallel BOIN approach, and 35% for the CRM shift model design [Figure 1(a)]. The probability of correctly selecting at least one true MTD is 40% for the parallel 3 + 3 approach, 60% for the parallel BOIN approach, and 64% for the CRM shift model approach [Figure 1(b)]. The probability of selecting both true MTDs is 5% for the independent 3 + 3 algorithms, 12.5% for the independent BOIN designs, and 38% for the CRM shift model [Figure 1(c)]. Finally, the probability that the parallel 3 + 3 approach reverses the MTD between each row is 31.5 and 0% for the CRM shift model [Figure 1(d)]. The reversal percentage is 30.3% for the parallel BOIN approach. These results are consistent with our findings in the previous section on a smaller number of dose–toxicity curves.

## Conclusions

More complex research questions are being posed in early-phase oncology clinical trials, necessitating design strategies tailored to the contemporary study objectives. Rule-based methods intended for MTD-based dose-finding are inflexible and cannot account for additional complexity presented by contemporary early development trials [19,20]. We have described an adaptive design strategy for a proposed early-phase trial concurrently evaluating the safety of a hematopoietic progenitor kinase-1 inhibitor (Agent A) as a single agent and in combination with an anti-PD-1 agent in patients with advanced malignancies. Although the design is presented using a specific example, the approach described could be generalized to other drug combination structures. For instance, Wages [9] applied the methodology described in this paper in settings with four dose levels in each of two rows and six dose levels in each of three rows. These settings studied the use of multiple models representing all possible shifts between dose levels in each row. Furthermore, our approach can connect with the vast body of published designs for determining an optimal administration schedule for each agent, incorporating efficacy outcomes in the dose selection model, and considering any potential ordering of toxicity and/or efficacy in subgroups of participants (see Lin *et al*. [21] for a recent example).

This manuscript highlights an example of a novel design application to augment future innovative design implementation and demonstrate adaptive designs’ flexibility in satisfying dynamic design conditions. The FDA and others encourage more innovative approaches [22,23]. The description of the general design strategy and thought process for implementation is the information that improves understanding, acceptance, and approval of novel designs [24,25]. Unfortunately, details of study designs often are not found on sites such as clinicaltrials.gov so that modern clinical trials lack the transparency needed to support the timely implementation of novel methods. Thus, displays of current trials that use novel methods are needed to overcome barriers of infrequent implementation of innovative design strategies in early-phase trials, so we believe this work can aid in the uptake of novel design use. In addition, given the often-lengthy timeline between study concepts and protocol completion, it is valuable to present design considerations with broad application. It is worth noting that even after study completion, journals do not require complete protocols as supplemental material for dose-finding trials, and final clinical trial publications do not have sufficient room to describe the details of novel designs. Therefore, we feel the message that novel methods are being used in clinical practice is timely and important. This support for adaptive strategies will augment efficient early-phase trial design in drug combination studies. Well-performing dose-finding methods can have a great impact on the drug development process [18].
